# The Institutional Library

**Published:** 1920-07-03

**Authors:** 


### THE INSTITUTIONAL LIBRARY.

### A LORD MAYOR'S DIARY, 1906-07.*

The author of this book is already known as a hospital founder in connection with the Alton Cripples Hospital for children suffering from tuberculosis of the joints, which bears his name. It was perhaps the chief work of his year of office, when Lord Mayor of London fourteen years ago, to establish his cherished institution, which has since achieved considerable reputation; so much so that tuberculosis medical officers ambitious to master their profession go there to study surgical tuberculosis. Alton Cripples Hospital is a place of teaching as well as a place of treatment. But the story of Sir William Treloar's efforts on its behalf are the best part of this genial and instructive book. It contains his official diary, a brief reference to the varied functions which succeeded each other day by day; and some words on the prominent persons whom he met. The charm of the whole lies in the fact that here is a Lord Mayor of London who thoroughly enjoyed every incident in his year of office, and is not ashamed to say so; who is at once the spectator of the scene wherein he played his part, and is content to record the feelings excited by it. Here is in fact a human peep behind the scenes. We know what a Lord Mayor feels on the day of the Lord Mayor's procession! what is the sensation of sitting in that coach, and just how tired is its occupant at the end of its long and swaying journey. We receive an amusing, and not undignified, account of one who went to Windsor to be knighted, and how the accolade was given, and what the Royal footmen did when the two knights left Windsor for home. All this is interesting and human; and those who have no access to official life will enjoy the story of the conversations and arrangements that preceded the Lord Mayor's official visit to Berlin. Sir William, like a sensible man, appreciates the trappings of his great office. He has a sense of history and tradition, and knows that etiquette and convention are valuable because these alone can make an official occasion pass off with comfort, because, without precedence and the guidance and order which precedence gives, a large assembly becomes a pushing throng of sightseers; whereas the manner of reception and public hospitality are arts which can be practised to perfection only by those who have been bred in their tradition. If the Mansion House is famous for its hospitality and the splendour of its public welcomes to official guests, it is due to this tradition. Nothing can replace that or remake it; and the tradition is supremely worth preserving. This book, therefore, in its proof of this is an education to read, and does, indirectly, a great service to the Mansion House. With one of the author's suggestions we heartily agree—namely, that Sir W. J. Soulsby, who has been Permanent Secretary to the Lord Mayors for a generation, should follow suit, and give the world the benefit of his recollections also. It takes a hospital man, perhaps, to recognise the value of tradition in any institution's life, and Sir William recognises it so well that we are sincerely grateful for his example.

* To which is added the Official Diary of Micajah Perry, Lord Mayor 1738-9. By Sir William P. Treloar, Bart. (London: John Murray. Price 10s. 6d. net.)

### THE REALITIES OF WAR.*

SIR PHILIP GIBBS, the well-known war correspondent, has done a good service in the publication of a book under the above title. In it he has endeavoured to place before his readers the horrors of war in their naked ugliness, unrelieved by any of the glamour and romance which are associated by poets and historical writers with the martial exploits of men and armies. With the exception of the men in the actual fighting-line and the medical men who attended to the wounded the actual horrors are little known, so that a glimpse such as is here given may have a salutary effect on those who talk lightly of fighting and going to war.

The author has apparently formed the opinion that modern war, with its gases and high explosives, is more horrible than war before the days of such inventions; but the human body can only appreciate a certain amount of suffering, and those who have read details of the battles of our forefathers may well doubt whether wars are worse now except in the multiplication of suffering due to the gigantic numbers employed in a modern battle. It is, however, very doubtful whether even an actual experience of the full horrors of a modern war such as the French must have experienced will prevent war again on a large scale, since it seems to be a provision of nature that pain is soon forgotten, else life would be one long agony.

In this book the writer seems to have been overwhelmed by the horrors, and to have been unable to propound any tenable theory of the meaning of it all. The slaughter of youth seemed senseless, and in his detestation of it all he is inclined to be—we think unjustly—severe on those older members of the community who could take no part in the fighting. He sees little of the heroism and self-sacrifice, and is impatient with the shortcomings of human nature.

In the more congenial task of trying to find some good arising out of the Great War—so far we have looked in vain through the pages of the "Realities of War." Looking back in history we find that most great advances have occurred as the result of disasters with their accompanying sacrifices. So close is the association that it seems to be a law of nature that some sacrifice must take place as an antecedent to reform and advance of the human race. Modern preventive medicine is the result of disastrous epidemics in the middle ages. Preventive medicine has made advances in five years that under peace conditions would have taken at least twenty-five. Miners and persons overwhelmed with conflagration and noxious gases will bless the many inventions against these enemies invented as the result of gas and flame attacks. Sailors and passengers will profit no less by the numerous devices against the U-boat menace, and sufferers from nervous disorders will find physicians with a much deeper acquaintance of their trouble. Workers of all sorts, especially in factories, will find their health more cared for as the result of our experience in the manufacture of munitions. Many examples of good out of evil could be enumerated should time and space permit.

* *Realities of War.* By Sir Philip Gibbs. (London: Wm. Heinemann, 21 Bedford Street. Price 15s. net.)

**The Institutional Library**—(*continued*).

**Altitude and Health.** By F. F. Roget, *Privat-docent* University of Geneva. Small quarto, pp. 186+vii. (London : Constable and Co. 1919. Price 12s. net.)

This book, as both the author and Sir William Collins explain in prefaces to it, originated in a course of three Chadwick lectures delivered in London by Professor Roget in the fateful year 1914. Of course it refers particularly to the Alps, and is just the sort of thing an Oxford or Cambridge don would like to take away with him on a "winter sport" expedition. Professor Roget is an enthusiastic highlander, or, to be more precise, a "6,000-feet man." Summer dew is uninteresting, but the collection of rime crystals upon snow a fine sight—he says hardily in more senses than one. He is up to date in his physiology, and even in the histo-pathology of heliotherapy, although, like Chadwick himself, not a medical man. One suspects, though, that from the original manuscript, which has lain by since 1914, a few German names have been judiciously removed. It is certainly a work likely to be useful to either tourist or *poitrinaire*, as also to their advisers, and mixed with the informative matter there are streaks of "middle-aged fun." We do not know the author's years, but humour expressed in a foreign language seems, like Lotos land, to be "always of an afternoon." There is a suspicion of book-making about the production, too, but it would be useful enough upon one's shelves if a consumptive had to be sent to Switzerland. The modern patient wants to know so much.

**Malaria at Home and Abroad.** (Illustrated.) By S. P. James, M.D., D.P.H. (Lieut.-Col. I.M.S. retired). (London : John Bale, Sons and Danielsson, Ltd., Great Titchfield Street, Oxford Street, W. 1. Price 25s. net.)

There have been so many books on this subject, both good and bad, published since the war period that it is at first sight difficult to welcome the appearance of yet another, but in this instance, coming from the pen of so experienced an authority, we can heartily recommend his work as one of the clearest and most practical expositions of the malarial question that it has been our privilege to read. The whole subject is tackled with a nice degree of appreciation for the relative importance of the non-medical point of view. Two chapters are of particular interest—namely, "A Malarial Survey in England" and "On Malaria in Children."

**The Industrial Clinic.** By Several Writers. Edited by E. L. Collis, M.D., M.R.C.P. Pp. 239. (London : John Bale, Sons and Danielsson. 1920. Price not stated.)

Dr. Collis, in his introduction to the above manual, claims that much of the material presented is taken from rather inaccessible Government publications, and is of unrecognised value. One does not find, however, much of relevance that would not suggest itself to the capable physician or administrator given the job of caring for factory workers, especially after a little experience. The first paper of the series is as good as any, written with judgment and sound sense by a Manchester certifying surgeon. Another, by a layman, on selection of workers, is redolent of practical deduction too; while a lady physician's is notable as citing by name Lord Beaconsfield, Mr. Taft, Mr. Kipling, King Solomon, the philosopher Locke, and the wrestler Hackenschmidt. In twenty years' time the subject will have developed a great deal, although not perhaps in the expected direction. Certainly "selection of workers" will take on a vast importance. By that time the tone of the present volume, slightly expressed as that is, will probably have become *rococo*. One contributor speaks of working-class parents seeking to have their children employed, from "greed." Might he not have said more correctly "need"? Should he not, at all events, have also used some warmth of expression when chronicling the facts that the law permits boys of fourteen to do night-work, and to be exposed to fumes that rot their teeth? As a last question, is he not like the reviewer—who, sooner than let a child of his own be treated so, would see all the eminent persons of the day in a variety of painful, humiliating, and dangerous positions?

**The Doctor's Manual.** By A. Herbert Hart, M.S. (John Bale, Sons and Danielsson. 1920. Pp. 256. Price 10s. 6d. net.)

This is stated to be the fourth, or *post-bellum*, edition of a book which contains the substance of a previous work entitled "How to Cut the Drug Bill." In many respects the book tramples on professional ethics and susceptibilities, and it is surprising to find that Mr. Frank Kidd has allowed his name (and his photograph as a frontispiece) to be used in connection with it. First come sections on the use and preparation of stock mixtures; they may be useful in the interests of economy, but (especially in conjunction with a later section) are apt to encourage the less conscientious in that penny-in-the-slot treatment which is the bane of National Health Insurance and club practice. Next follows a section on "Anæsthetics." It deals exclusively with local anæsthesia, and can be divided into three parts, which are, to a certain extent, mutually contradictory, and appear to have been separately composed at different dates. At the end of the chapter is a brief notice, from which it appears that some part (or the whole) of the text of it is a reprint of a paper published in America before the war. Next are sections which are the most unsatisfactory of all, because they lay the author open to what may be a quite baseless suspicion that his enthusiasm for certain manufacturing firms has been stimulated by their undisguised advertisements which appear before or after Mr. Hart's pages. There are other objections to the whole style and manner of this book, which is calculated to make any sensitive member of the medical profession hang his head.

**Advice to Consumptives.** Home Treatment, After-Care, and Prevention. By Noel Dean Bardswell, M.V.O., M.D., F.R.C.P. Second edition. (London : A. and C. Black, Ltd. Pp. 153. 3s. 6d. net.)

Dr. Bardswell rightly lays stress on faithful adherence to rule and regulation for varying periods after leaving the sanatorium. Improved appearance and a general sense of well-being conspire to delude patient and friends into a false sense of security, which in its turn tends to produce slackness in the carrying on of treatment. When the author goes on to state that actual cure may be assumed after two years' freedom from all signs and symptoms, he is on more debatable ground. Surely if any word should be used with bated breath in connection with pulmonary tubercle it is the word "cure."

Only one other criticism of this valuable book occurs to us. The chapter on "Food" might well have been revised in view of the totally different conditions existing to-day as compared with ten years ago when the previous edition was published. Many people simply cannot regulate their purchases by the advice and suggestions given. A serious attempt to bring this chapter under control of present-day conditions would not only have been of real benefit to many harassed housekeepers, but would have obviated an easily received impression that the book as a whole is simply a reprint of the first edition.

